# Lean Unet: a compact model for image segmentation

**DOI:** 10.1007/s11548-026-03699-9

**Published:** 2026-07-02

**Authors:** Ture Hassler, Ida Åkerholm, Marcus Nordström, Gabriele Balletti, Orcun Goksel

**Affiliations:** 1 Dept of Information Technology, Uppsala University, Uppsala, Sweden; 2 RaySearch Laboratories, Stockholm, Sweden

**Keywords:** Pruning, Network compression, Information bottleneck, Medical image segmentation

## Abstract

****Purpose**:**

Unet and its variations have been standard in semantic image segmentation, especially for computer-assisted radiology. Current Unet architectures iteratively downsample spatial resolution while increasing channel dimensions to preserve information content. Such a structure demands a large memory footprint, limiting training batch sizes, and increasing inference latency. Channel pruning compresses Unet architecture without accuracy loss, but requires lengthy optimization and may not generalize across tasks and datasets.

****Methods**:**

By investigating Unet pruning, we hypothesize that the final structure is the crucial factor, not the channel selection strategy of pruning. Based on our observations, we propose a lean Unet architecture (LUnet) with a compact, flat hierarchy where channels are not doubled as resolution is halved.

****Results**:**

We evaluate on a public MRI dataset allowing comparable reporting, as well as on two internal CT datasets. We show that a state-of-the-art pruning solution (STAMP+) mainly prunes from the layers with the highest number of channels. Comparatively, simply eliminating a random channel at the pruning-identified layer or at the largest layer achieves similar or better performance. Our proposed LUnet with fixed architectures and over 30 times fewer parameters achieves performance comparable to both conventional Unet counterparts and data-adaptively pruned networks.

****Conclusions**:**

The proposed lean Unet with constant channel count across layers requires far fewer parameters while achieving performance superior to standard Unet for the same total number of parameters. Skip connections allow Unet bottleneck channels to be largely reduced, unlike standard encoder–decoder architectures requiring increased bottleneck channels for information propagation.

**Supplementary Information:**

The online version contains supplementary material available at https://doi.org/10.1007/s11548-026-03699-9.

## Introduction

Semantic image segmentation is a fundamental processing step in many computer-assisted interventions including surgical planning, guidance, intraoperative navigation, and radiotherapy—all requiring automatic, accurate, and efficient methods. Unet [1] is a neural network (NN) solution which has become the de facto standard for segmentation in most supervised learning settings. It is also commonly used as a backbone in other NN settings such as for contrastive learning [2], detection/density estimation [3], diffusion models, and generative adversarial networks, especially for processing medical images, where context is crucial. In contrast to natural images, where observed objects are rarely attached to their background, imaging of anatomy is strictly context dependent, *i*.*e*., the location and morphology of organs are relatively fixed. Unet incorporates such contextual information with its multi-level-of-detail approach, by distilling contextual info at coarsening NN layers of its encoders, while employing such information for refining resolution in its decoder layers. In contrast to conventional encoder–decoder architectures, skip connections in Unet facilitate the merging of contextual and resolution-specific information for precise pixel-resolved outputs.

Traditionally, deep learning has relied on the manual design of carefully constructed and empirically validated neural network architectures, often employing over-parameterized models to capture all potentially relevant information. To avoid overfitting, one then requires large datasets as well as several optimization and regularization tricks such as Monte Carlo dropout at training time, optimized hyper-parameterizations, early-stopping, elastic weight regularization, among others. From a function approximation point of view, by Occam’s razor, and in practice, a network should be *as complex as needed while as simple as possible* to provide an efficient, low-footprint representation that also prevents overfitting. Neural Architecture Search (NAS) [4] is an automated machine learning (AutoML) approach for finding optimal NN architectures by iterating over possible options using often black-box optimization approaches such as evolutionary search, reinforcement learning, and Bayesian function approximation. Pruning is an alternative technique aiming at network compression by reducing NN model sizes via removing neurons or parameters that are selected based on various criteria [5]. For Unet in particular, a recent method [6] proposes iteratively pruning the neurons (*i*.*e*., convolutions/channels) that produce the lowest activations during training. This generates much smaller (sub)networks of the initial Unet, while also reducing overfitting, which is a challenge especially for smaller training sets. Further, ad hoc compact versions of Unet have been suggested [7, 8] though without principled approaches or optimality guarantee in devising their architectures.

In this work, we utilize network pruning as a tool to help identify optimal Unet architectures. We closely examine gradual channel pruning in Unet, focusing particularly on the temporal evolution of channel selection during pruning. We experimentally study the relative importance of the chosen specific channels and their locations in the architecture, with key comparisons to random and systematic (hand-crafted) pruning baselines, while interpreting these in the context of Lottery Ticket Hypothesis (LTH) [9] and synaptic saliency [10]. From our experiments, we conclude that such Unet pruning optimizes its architecture, rather than choosing channels with specific weights/activations—an observation that corroborates with some literature while contrasting with others. Inspired by the asymptotic convergence of the NN architectures in our experiments, we propose a Lean Unet (LUnet) architecture with a fixed number of channels per block. We show LUnet to perform comparably to or better than both pruned networks, which require complex, data-dependent processes, as well as corresponding Unet baselines, despite having over 30 times fewer parameters.

## Methods

### Neural network compression

Network pruning can yield models capable of performing on par with or superior to their dense counterparts [5, 9] while using less than half of the original parameters, highlighting the substantial redundancy present in state-of-the-art deep neural networks. Some approaches aim for one-shot pruning prior to training, to remove structurally unimportant connections; for instance, SNIP [11] proposed a saliency criterion based on gradient and weight magnitudes at the initial training step. The Lottery Ticket Hypothesis (LTH) [9] claims that certain subnetworks may perform particularly well due to their fortunate random initialization. To utilize this, a NN is trained to convergence, the weights with the smallest magnitudes are removed, and the pruned subnetwork is retrained with its *original* initial weights—in a loop repeated iteratively until a desired sparsity is reached. LTH was later challenged by Liu et al. [12], who showed that an arbitrary initialization may generate similar results, indicating that the key factor is the architecture and not the initialized weights.

Synaptic saliency is defined in [10] as a group of pruning criteria $\frac{\delta R}{\delta \Theta }*\Theta$ where Θ is the network parameters and *R* is a function of the network output such as loss. Having demonstrated the conservation of cumulative synaptic saliency between the input and output of a layer, the authors argue that the individual neurons of a wider layer should have lower saliency on average. Their method (SynFlow) hence is more likely to preferentially prune from larger layers.

**Figure:**
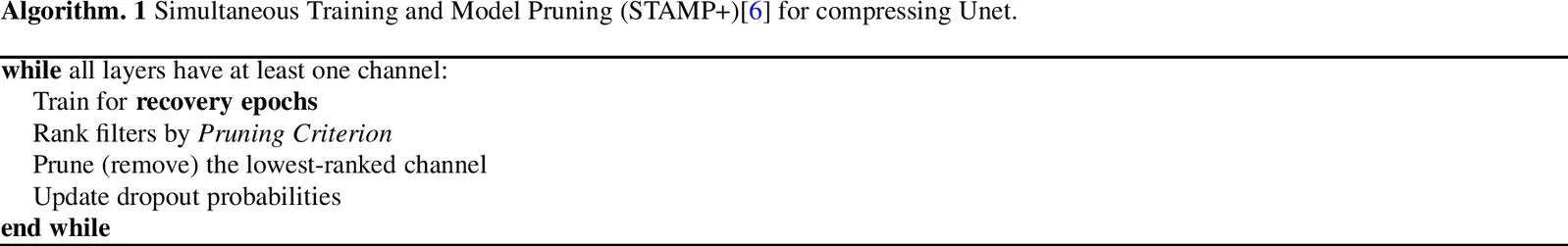

**Fig. 1:**
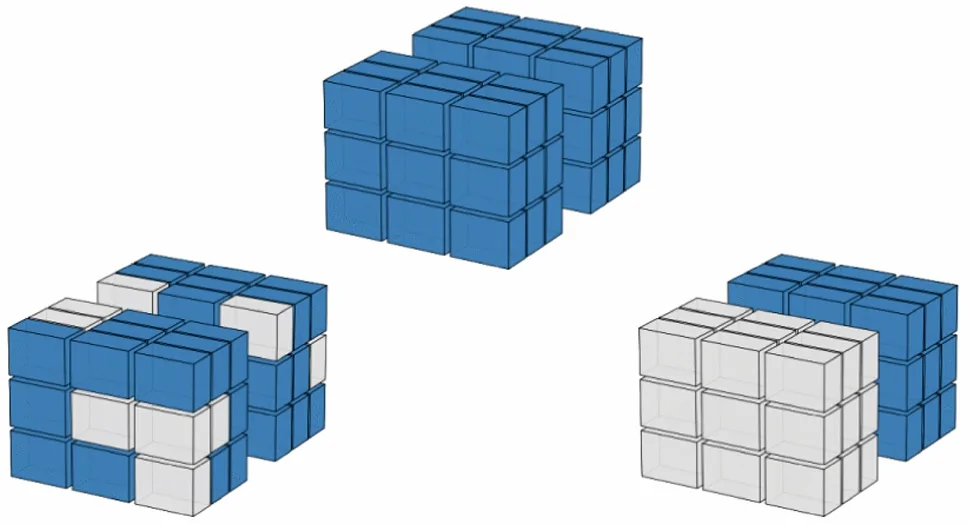
Visualization of pruning strategies on two convolution channels with 3x3 filters and 3 channel input: dense (top), unstructured parameter pruning (left), and structured channel pruning (right)

Pruning can be performed in a structured or unstructured manner, as illustrated in Fig. 1 for a convolution layer. Unstructured pruning removes weights (connections/synapses), thus changing the NN structure, while structured pruning removes neurons (equivalently, convolutions/channels in CNNs) hence preserving structure [13, 14]. Several variants of structured pruning have been suggested for Unet compression [6, 15, 16]. Note that structured pruning of a neuron directly reduces the computation time and memory footprint for activations, whereas unstructured removal of weights may still require the computation of corresponding convolutions (with some zero filter weights), thereby not necessarily reducing computation or storage—unless the utilized low-level libraries have support for related sparse operations.

### Gradual channel pruning

Some pruning methods wait until the convergence of training to make any pruning decisions, since such a state provides certain theoretical properties, *e*.*g*., gradients being negligible. An iterative application of such pruning, however, requires huge resources, since the network needs to be retrained after each pruning step. Alternatively, *gradual pruning* aims to compress networks during training, thus learning simultaneously both the NN structure and the weights. For instance, FGGP [17] ranks gradients and weight magnitudes to prioritize parameters to prune. Structured pruning of convolution channels in a gradual fashion is known as gradual channel pruning. This can use different pruning criteria such as LASSO regression [18], discrimination-aware loss [19], or mean activation magnitude [6]. Simultaneous Training and Model Pruning (STAMP) [6] is a gradual channel pruning method specifically for Unet. It alternately trains for a fixed number of (so-called, *recovery*) epochs and then removes the channel with the lowest normalized activations. For robustness of training to the disappearing channels, STAMP+ [6] employs a targeted channel-wise dropout strategy with higher probabilities for channels that are more likely to be pruned soon. Its algorithmic prototype is shown in Alg. 1, as also used as a baseline in our work.

### Unet architectures

Inspired by encoder–decoder architectures, typical Unet [1] doubles feature channels at each encoder downsampling stage in order to preserve information content. This principle is also inherited in any newer Unet versions and improvements such as nnUnet [20] and Unet++ [21], as well as in Unet-like architectures utilizing transformers such as Swin-Unet [22] and UCTransNet [23], and transformer-inspired convolutional architectures such as MedNeXt [24]. Such doubling with depth is indeed a primary factor inflating Unet parameter sizes. Nevertheless, a key differentiator of Unet from simple encoder–decoder structures is the skip connections. Since these enable direct information flow from encoders to decoders at corresponding levels, it can be argued whether channel doubling is really essential in a Unet architecture. In this work, we study and test this hypothesis based on observations on gradual channel pruning and its variants.

### A Lean Unet (LUnet) architecture

As seen later in our results, we test switching the STAMP+ pruning to a random version while keeping its architecture, as well as preferentially pruning the largest blocks (layers) regardless of their weights. Based on our findings, we conclude as a rule of thumb that minimized Unet architectures for segmentation can function successfully with channel sizes equal across blocks, especially at coarser levels since information preservation is not a concern thanks to skip connections. This leads us to the idea of using the limit case of such pruning, as a manually defined Unet substructure having a fixed number of channels at every block/level. We call this structure as Lean Unet (LUnet). LUnet provides a small model without the overhead of pruning and involves no data-specific adaptation, both of which make it much less likely to overfit data.

### Evaluation and datasets

Analyses and evaluations were conducted on three datasets: Hippocampus segmentation in MRI with a harmonized protocol (HarP) [25], which allows us to compare the results with baselines on a public dataset. Two internal datasets for submandibular gland (SG) and tracheal tree (TT) segmentation, respectively, complement this with evidence on CT images, with the latter dataset also providing a multi-label segmentation setting. Dataset details are listed in Table 1. We trained baseline Unet structures conventionally from initial random weights. We also gradually pruned them during this same process using different strategies described later in the experiments. We ran such gradual pruning to extreme sparsity until model collapse and reported the maximum Dice values observed during training. This was chosen for consistency with baseline STAMP+ reporting and to show baselines in their best-case setting. For evaluation, we used the Dice similarity metric and average Dice across labels for the TT dataset.

**Table 1:** Summary of dataset properties and the hyper-parameters used

Dataset	HarP	Submandibular Gland (SG)	Tracheal Tree (TT)
Modality	T1 MRI	CT	CT
Image size (voxels)	64x64x64	128x128x128	128x128x128
Task (labels)	Hippocampus	Submandibular Gland	Trachea, Bronchus L/R, Carina, Trachea extension
Train→Test Splits	200→70; 50→220	222→56	64→16
Batch size	16	1	1
Recovery epochs	5	1	1
Levels in Unet	5	4	4
Convolutions per block	2	3	3
Channels-per-block at top layer	4	24	24
Learning rate	0.01	0.001	0.001

### Implementation

For STAMP+ [6], we used the code^1^ provided by its authors, by fixing a memory leak that initially prevented us from training on large images and longer training episodes. We made minor modifications for compatibility with Python 3.11 and PyTorch 2.2, without altering any functionality. For comparability, the hyper-parameters for the HarP dataset were chosen to closely replicate the STAMP+ settings [6] with a relatively low number of convolution filters in the top (initial) Unet level, which enabled larger batch sizes. For the CT datasets TT and SG, we employed a much larger 3D Unet baseline architecture that is representative of typical Unet model sizes used in production and in the literature. This allowed a batch size of only one image volume to fit in memory. For the CT datasets, preliminary experiments indicated that a recovery epoch setting of one was sufficient for results comparable to higher settings. A summary of varying hyper-parameters is provided in Table 1.

We used L$_2$-norm of channel activations as the pruning criterion as suggested in [6], and a base probability of 5% for targeted dropout. All models were optimized using the Soft Dice loss function and the ADAM optimizer with momentum parameters of $\beta _1$=0.9 and $\beta _2$=0.999. Due to the smaller batch sizes of SG and TT, a smaller learning rate was used. All experiments were run on an NVIDIA RTX A5000 GPU with 24 GB of VRAM. Reported FLOPs were computed using Python calflops.

As modern baselines, we evaluated MedNeXt [24] and Unet++ [21]. For both, we took the official repository code and integrated it into our framework, standardizing preprocessing and data augmentation. Lean Unet++ was implemented by excluding channel doubling after downsampling in Unet++.

## Experiments and results

On the three datasets, we conducted experiments under 4 settings, where HarP was tested with two different train–test splits, see Table 1. Each experiment was repeated three times using random initializations, reporting their median and MAD (median absolute deviation). In the interest of space, we present the analysis figures below only for HarP 200→70 split, with additional analyses provided in Supplementary Material.

### Gradual channel pruning

**Fig. 2:**
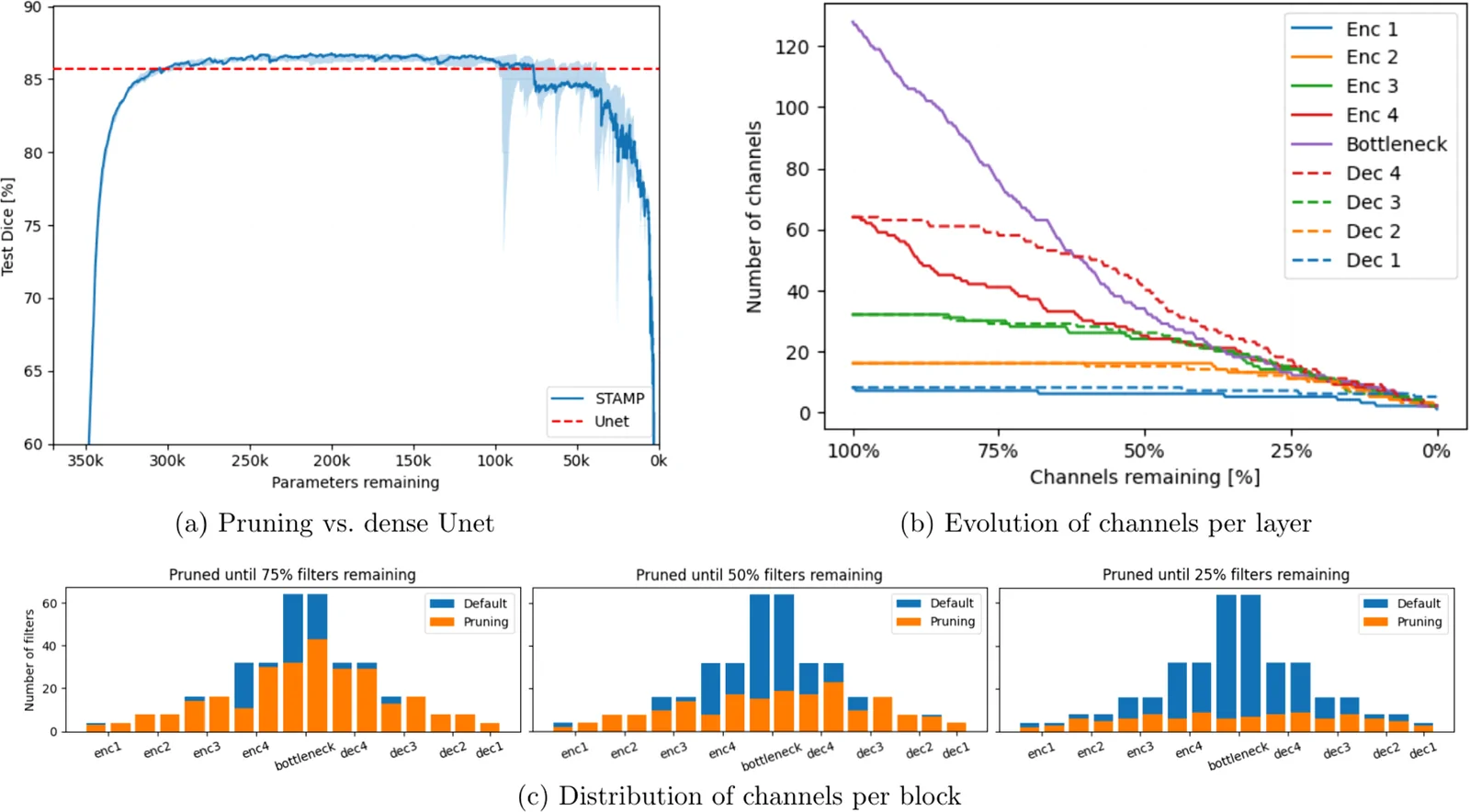
Pruning on HarP for 200→70 train–test split. (**a**) Test Dice score evaluated during training with STAMP+ set compared with the dense Unet baseline (Unet$_{100\%}$). (**b**) Remaining channels at each Unet encoder–decoder subpart during pruning. (**c**) Distribution of channels per block at three sample pruning steps when {75,50,25}% of total channels remain

We first trained a dense Unet baseline with an initial block size $N_\textrm{f}$=4 filters. We then gradually pruned its channels using STAMP+. Their comparative results shown in Fig. 2a corroborate the results presented in [6], where pruning is seen to outperform the Unet baseline across a wide range of sparsity values.

Analyzing channel sizes at each encoder–decoder layer as shown in Fig. 2b, Unet components with the largest channel counts are observed to be preferentially pruned first. Once the bottleneck and deeper layers are pruned down to channel sizes of shallower layers, only then the latter start being pruned as well. This causes the Unet architecture to slowly become flatter in channel size, starting with the deepest layers equalizing first. This pattern can also be observed in Fig. 2c which shows snapshots of individual channel sizes at each Unet block at three different pruning stages that correspond to when {75,50,25}% of total channels remain. This observation may be explained by the synaptic saliency [10] of a channel at a wider block or layer being on average lower, hence making those channels more likely to be selected by a chosen pruning criterion. This observation raises the important question: Is the architecture attained by pruning more pertinent than the actual channels selected for pruning? This somewhat parallels the arguments in [12] questioning the Lottery Ticket Hypothesis [9].

### Compressed architecture and parameter values

**Fig. 3:**
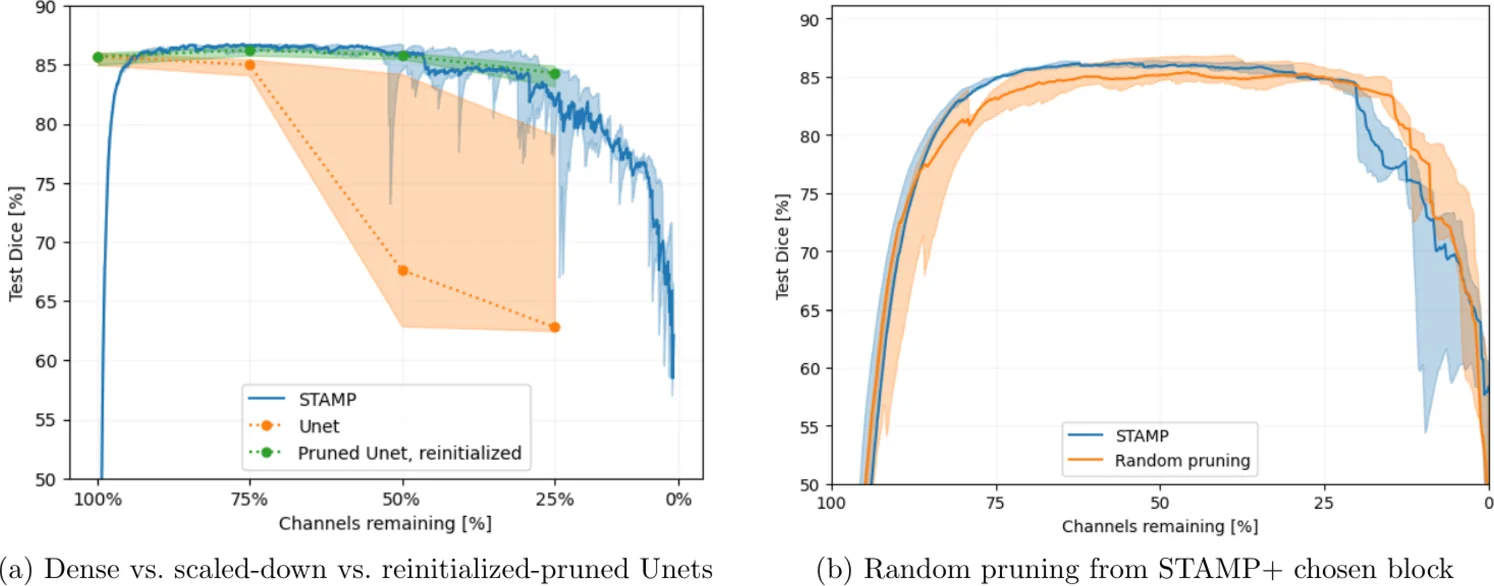
Dice comparisons of strategies. (**a**) Pruning compared to the baseline dense Unet$_{100\%}$ and linearly scaled-down models Unet$_{\{75\%,50\%,25\%\}}$ (orange) as well as equivalent-sized pruned networks after retraining from random initializations (green). (**b**) Pruning a random channel from STAMP+ determined block (orange), compared to pruning based on channel activations (blue). Curves show the median values, with min/max ranges of three repeated runs shaded

To test the above hypothesis, we took the network structures pruned above at {75,50,25}% channels remaining and retrained these from scratch by reinitializing their parameters with random values. These are shown in Fig. 3a

**Fig. 4:**
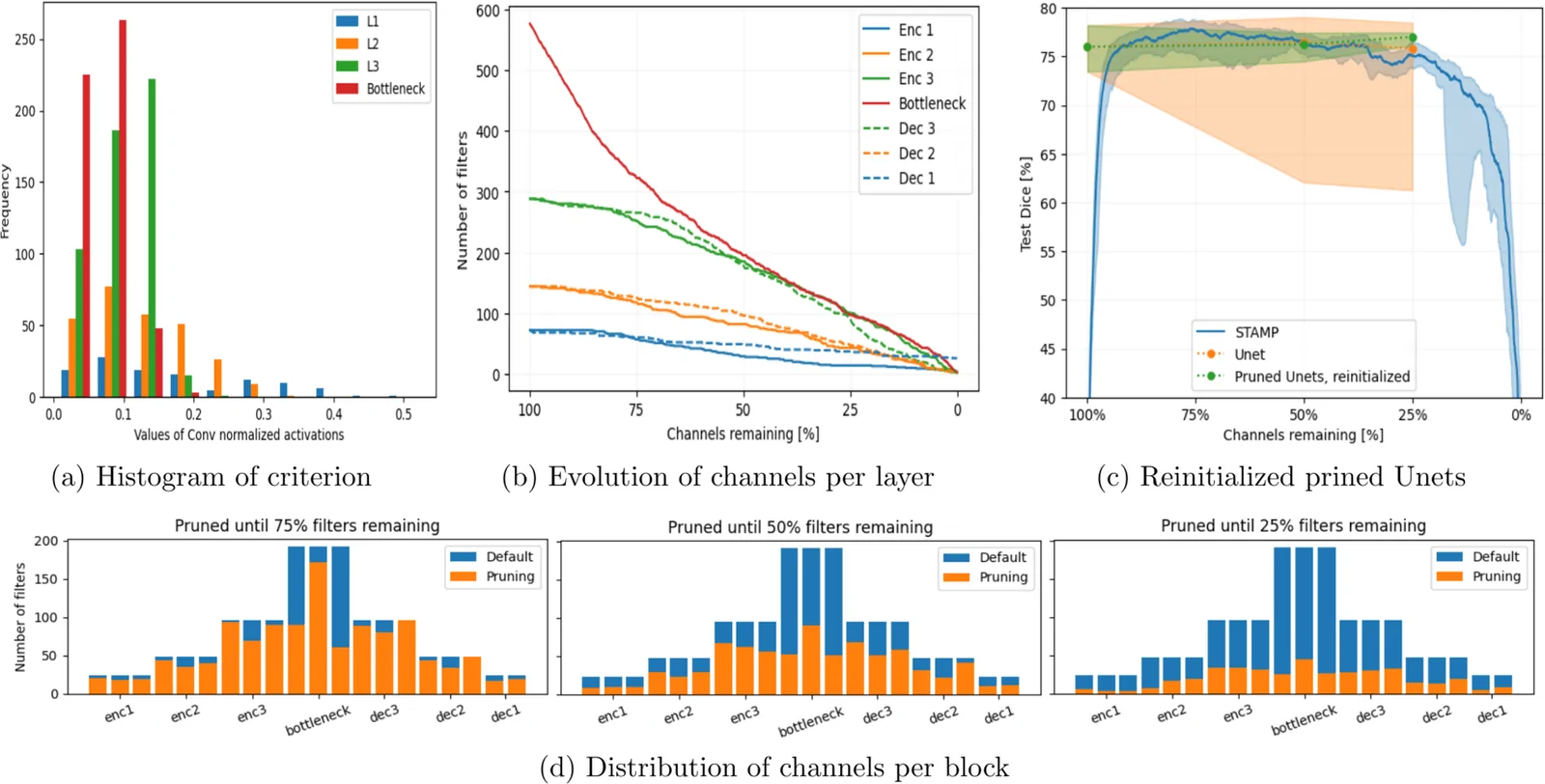
Sample results on the Tracheal Tree (TT) dataset: (**a**) Distribution of pruning criterion (normalized activations) per Unet level at 95% channels remaining. (**b**) Remaining channels at each Unet encoder–decoder subpart during pruning. (**c**) Pruning compared to the baseline dense and linearly scaled-down Unet$_{\{100\%,75\%,50\%,25\%\}}$ (orange) as well as equivalent-sized pruned networks after retraining from random initializations (green). (**d**) Distribution of channels per block at the pruning steps when {75,50,25}% of total channels remain

**Fig. 5:**
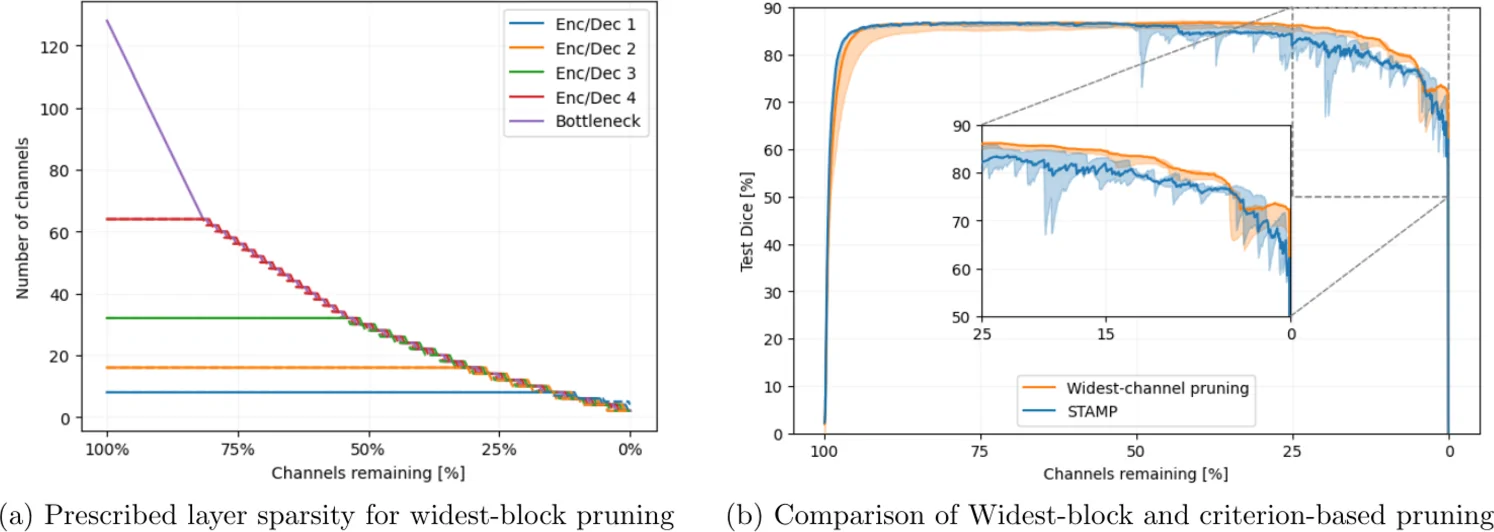
(**a**) Prescribed number of channels per Unet subpart for pruning always from the block with most channels, *i*.*e*., *widest-block pruning*. (**b**) Dice evolution of widest-block pruning and STAMP+

being non-inferior to STAMP+ pruning—and even superior at a high sparsity of 25%. Hence, in this particular setting, the network architecture seems to be more important than the actual weights during pruning. To check whether this advantage is simply due to less overfitting of a smaller network, we also tested corresponding Unet baselines (called Unet$_{\{75\%,50\%,25\%\}}$) whose number of parameters were scaled linearly at all blocks. These baselines, however, performed significantly worse than both pruning and pruned-and-reinitialized models, especially at higher sparsity. This indicates that the location of channels is indeed important, not merely the number of channels in total.

Since the channels of a block are symmetric and interchangeable considering random reinitializations in the above experiments, what is important must then be the overall architecture achieved by pruning, not the exact channels selected during it. We tested this hypothesis by pruning a random channel from the pruning-identified network block, *i*.*e*., not necessarily the exact channel chosen by the STAMP+ pruning criterion. In other words, we ran the pruning as normal and let it choose a channel, but then instead of removing that channel we removed a randomly sampled channel within that same block. This effectively achieves the same architecture and topology evolution as in Fig. 2b but with exact channels not being based on the pruning criterion. Figure 3b shows that such random pruning from the given block achieves similarly to or even better than targeted channel pruning of STAMP+, confirming that the pruned architecture is the key—not necessarily the weights. Some corresponding results for the TT dataset can be seen in Fig. 4.

### Systematic widest-channel pruning

To achieve flatter architectures similar to those observed above in Fig. 2b-c but without using any pruning criterion, we next tested a naïve approach of pruning a random channel from the largest block at each step. This results in a preset pruning schedule shown in Fig. 5a that is independent of training data and takes a direct shortcut to a flat channel distribution.

**Table 2:**
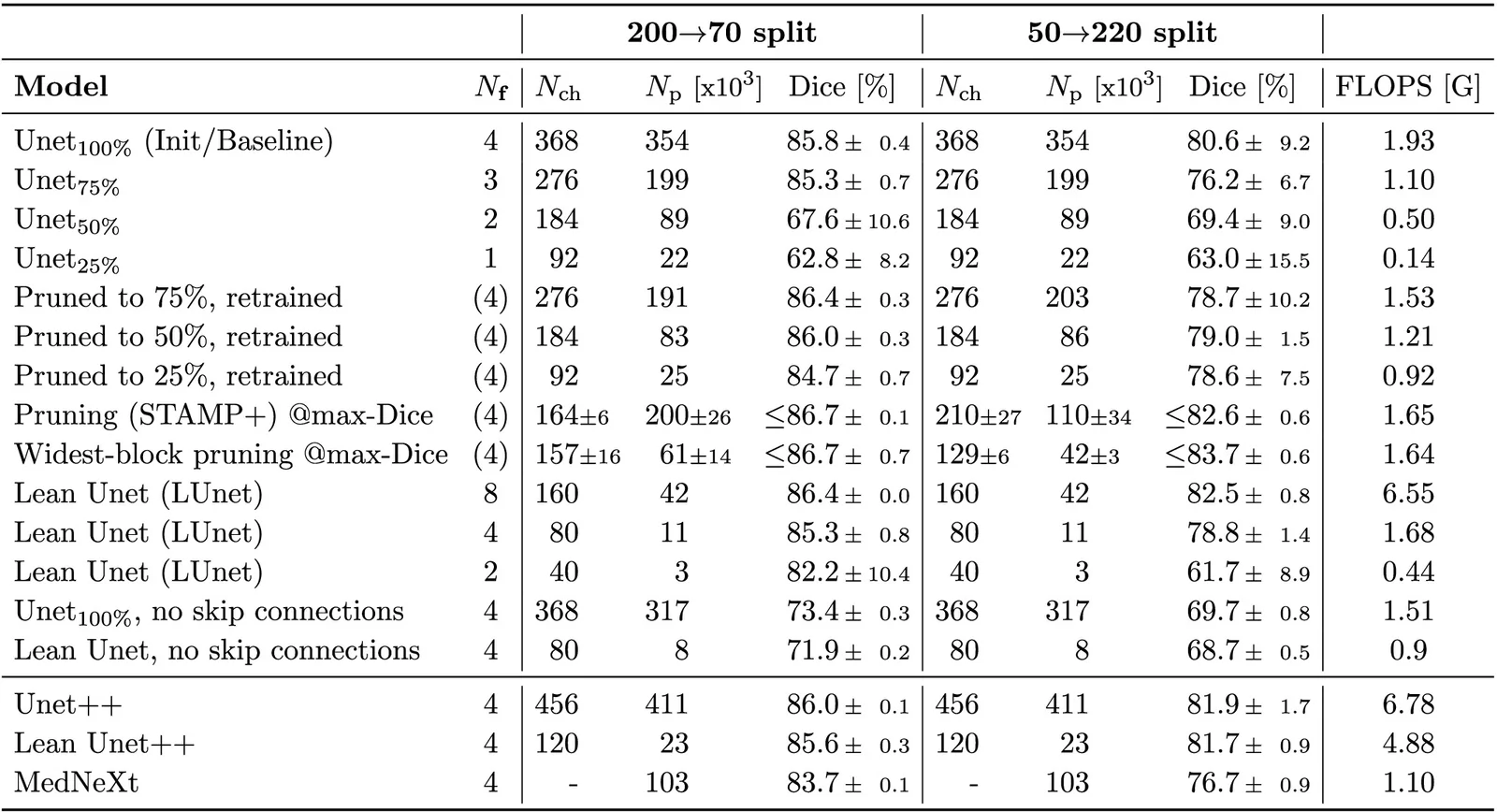
Median results (± median absolute deviation) for different Unets on the HarP dataset. Columns indicate $N_\text {ch}$ the total number of channels in the architecture, $N_\text {p}$ the total number of network parameters, $N_\text {f}$ the number of channels per block in the top Unet level (*f* in [6]), and FLOPs for the different architectures. (For pruned architectures, a mean value is listed.) Pruning may reduce $N_\text {f}$, so its value at initialization is indicated in parentheses (·)

**Table 3:**
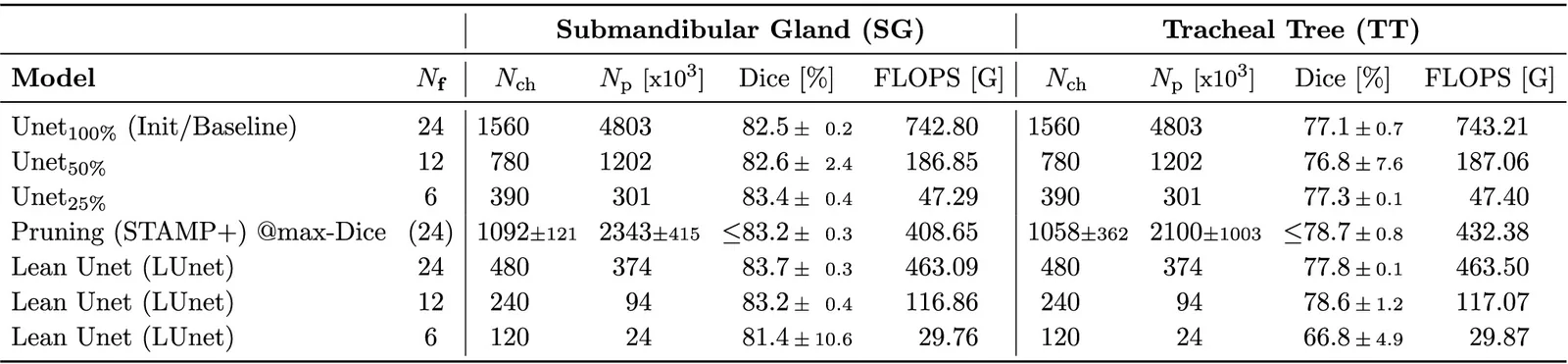
Median results (± median absolute deviation) for different Unets on the Submandibular Gland (left) and Tracheal Tree (right) datasets. Columns indicate $N_\text {ch}$ the total number of channels in the architecture, $N_\text {p}$ the total number of network parameters, $N_\text {f}$ the number of channels per block in the top Unet level (*f* in [6]), and FLOPs for the different architectures (for pruned architectures a mean value is listed). Pruning may reduce $N_\text {f}$, so its value at initialization is indicated in parentheses (·)

Strikingly, this simple approach outperforms standard STAMP+, especially at extreme sparsity values, as shown in Fig. 5c.

### Lean Unet

STAMP+ approximating a flat architecture and the above simplified widest-channel pruning performing similarly or superiorly to criterion-based STAMP+ lead us to our proposed solution Lean Unet (LUnet) with a fixed channel size in all blocks. Accordingly, the starting (top-level) channel size stays the same across all layers. LUnet results are tabulated in Table 2 comparatively with the variations presented above, for both train–test splits of HarP dataset. A comparison of LUnet and its baselines for the SG and TT dataset can be seen in Table 3. For pruning, performance is reported at the sparsity where it achieves its highest test Dice score, which is a highly biased metric indicating a best-case scenario unattainable in practice.

Furthermore, the large deviation in $N_\textrm{p}$ for these values indicates that the maximum Dice is achieved at different model sizes and hence pruning stages, which cannot be controlled nor predicted a priori, also indicating a lack of repeatability. Our results show that the proposed LUnet structure achieves similar performance, while being a fixed and data-independent structure, without requiring costly pruning operations to determine. With parameter counts much smaller than STAMP+, LUnet can achieve similar performance, *e*.*g*., 199 vs. 42 K parameters for HarP in Table 2. Moreover, conventional Unet architecture with linear scaling is seen to collapse around 88.8 K parameters (Unet$_{50\%}$), while our LUnet performs well even with a fraction of the parameter count (*e*.*g*., 2.8 K). With merely 42 K parameters, LUnet outperforms the largest Unet baseline (Unet$_{100\%}$) with 354 K parameters. Indeed, in the HarP setting for a fixed top-level block size $N_\textrm{f}$=4 channels, Unet contains 354/10.7>33 times more parameters than LUnet, and similarly 88.8/2.8>31 times more for $N_\textrm{f}$=2. LUnet reduces not only the number of parameters but also the computations as seen by the reported FLOPs. Comparing Unet and LUnet for the SG and TT datasets, LUnet uses 38% fewer FLOPs than the standard Unet for all tested top-level block sizes. Tests with the more modern Unet++ architecture show similar performance and computational savings, suggesting that the lean structure generalizes well. Further, experiments removing skip connections (Table 2) show a larger performance drop for LUnet than Unet, supporting that LUnet’s lean design relies more heavily on skip connections. Transformer-inspired MedNeXt baseline did not yield accuracy comparable to Unet or LUnet in our tested setting.

Inference time measurements using the PyTorch Benchmark module on SG/TT setting indicated that a 320$^3$ voxel clinical image taking 1.2 s to segment with the baseline Unet takes less than half that time with LUnet($N_\text {f}$=12), while also leading to better accuracy as reported in Table 3. Note that timing measurements may largely depend on the hardware, libraries, compiler, and other factors, hence we chose to report comparisons in FLOPs as common in the literature.

## Discussion and conclusions

By analyzing the distribution of channels during pruning and reinitializing pruned networks, we have shown that (1) pruning primarily removes channels in deeper layers and (2) reinitialized pruned models attain comparable performance when retrained. These insights motivated two simplified pruning strategies, both targeting the deeper layers first by pruning (1) some random channel from the block selected based on pruning criterion and (2) removing a random channel from the widest block at a given time. Both approaches achieve performance comparable or superior to original pruning, suggesting that pruning effectiveness depends more on the Unet structure it attains than on the specific channels it retains. We have proposed *LUnet*, a compact Unet variant with fewer channels in the bottleneck and deeper layers. A similar architecture was suggested in [7] as an ad hoc solution, which we herein derive using network pruning as an architecture optimization tool, while showing also comparatively to pruning and demonstrating on Unet++ as a modern variant. LUnet matches the performance of Unet baselines while using over 30× fewer parameters and reducing computations by up to a third. This result challenges the long-standing assumption that the number of channels should double after each downsampling—a design convention that has persisted since the original Unet architecture. With its much smaller structures, LUnet also matches STAMP+ performance as reported at its maximum test value—a reporting convention that we inherited from its original publication [6]. Note that such reporting of self-maximized test score is largely dependent on noise/stochasticity in training iterations and hence represents a highly biased upper-bound scenario which is not likely to generalize on a separate hold-out data; hence, we prepended these values with a “≤” in the tables. This is corroborated by the STAMP+ result reporting being an upper-bound for the retrained models at {25, 50, 75}% sparsity. For all models trained without pruning, such as LUnet, the reported test Dice is unbiased to test set since the models and weights are fixed once the training ends. Therefore, comparisons with pruned-and-retrained models are more representative of relative performance in real-world scenarios. Furthermore, pruning involves considerable computational overhead due to repeated model architecture updates and any additional recovery epochs. LUnet, in contrast, achieves competitive results with a fraction of the network size and in a data-independent, robust manner.

Smaller network sizes facilitate storage and transfer, such as for gradient update exchanges over network in federated learning. Faster segmentation models are beneficial in several computer-assisted intervention settings that require near real-time and/or hardware-constrained operation. For instance, online adaptive radiotherapy requires rapid segmentation with the patient on the treatment couch, where every minute saved can make a difference in terms of enabling a solution, minimizing patient motion, or allowing ensembles of models to run for accuracy boost and uncertainty assessment. Efficient models are crucial also for the near real-time constraints of surgical guidance, and in resource-limited settings such as capsule endoscopy.

## Supplementary Information

Below is the link to the electronic supplementary material.

Supplementary file 1 (pdf 316 KB)

## Data Availability

HarP dataset is accessible online. Private datasets are not available for sharing.

## Footnotes

1 https://github.com/nkdinsdale/STAMP.

## Code Availability

Code is available at https://github.com/tureture/LUnet
