# Supplementary material for: Lean Unet: a compact model for image segmentation
Source: Int J Comput Assist Radiol Surg. 2026 Jul 2;21(7):1593–601. doi: 10.1007/s11548-026-03699-9 (PMC13553731; doi:10.1007/s11548-026-03699-9)
Supplement: Supplementary file 1 [file 11548_2026_3699_MOESM1_ESM.pdf]

Lean Unet: A compact model for image segmentation — Supplementary Material —

Ture Hassler[1,2], Ida Åkerholm[1], Marcus Nordström[2], Gabriele Balletti[2], and Orcun Goksel[1]

[1] Dept of Information Technology, Uppsala University, Uppsala, Sweden
[2] RaySearch Laboratories, Stockholm, Sweden

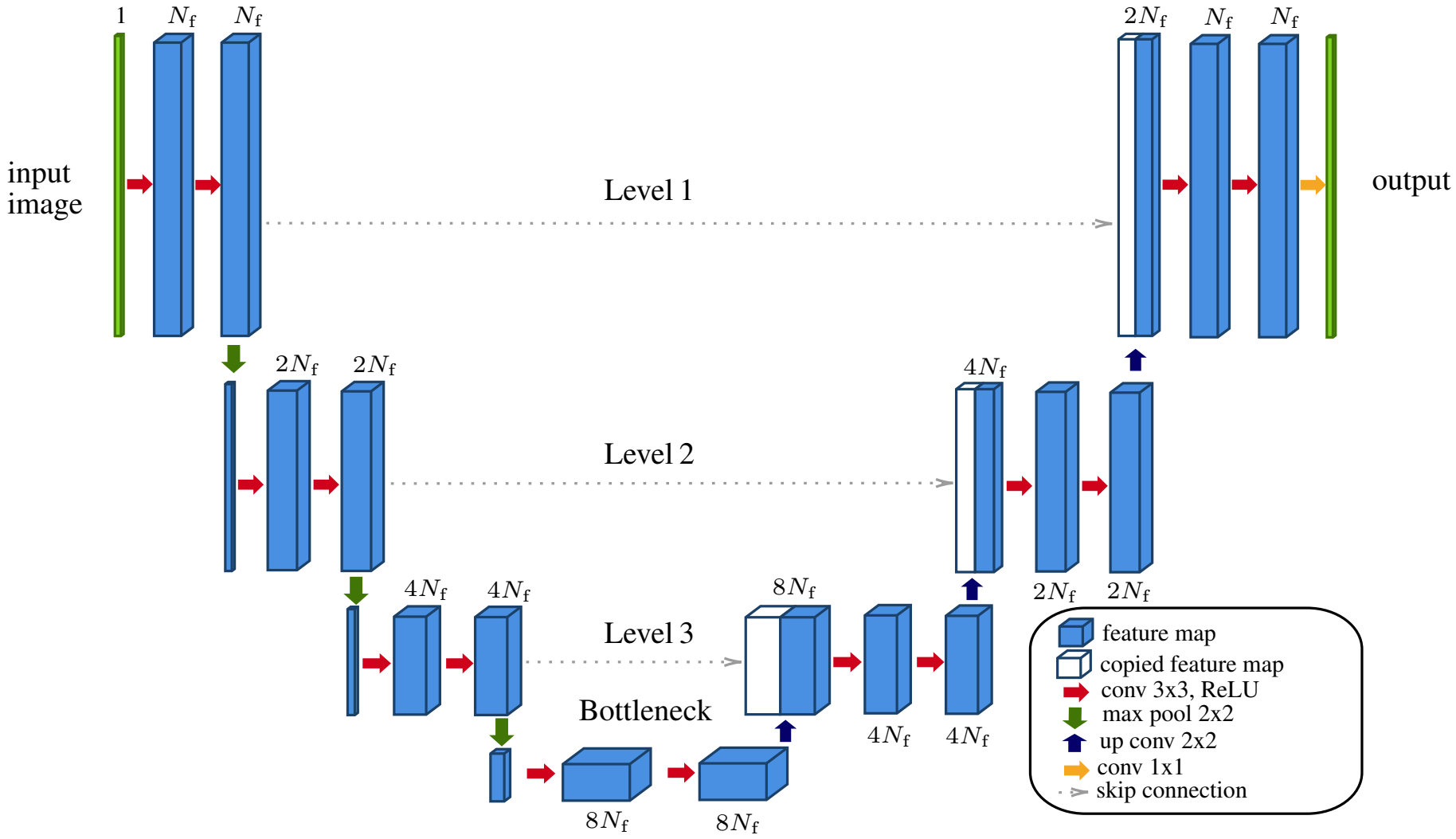

Fig. S1. Visualization of a regular Unet architecture, with N_f starting convolution filters, two convolutions per block, and a depth of four levels. LUnet architecture follows the same structure, except that the number of convolution channels per block remains constant across network levels rather than doubling at each successive level.

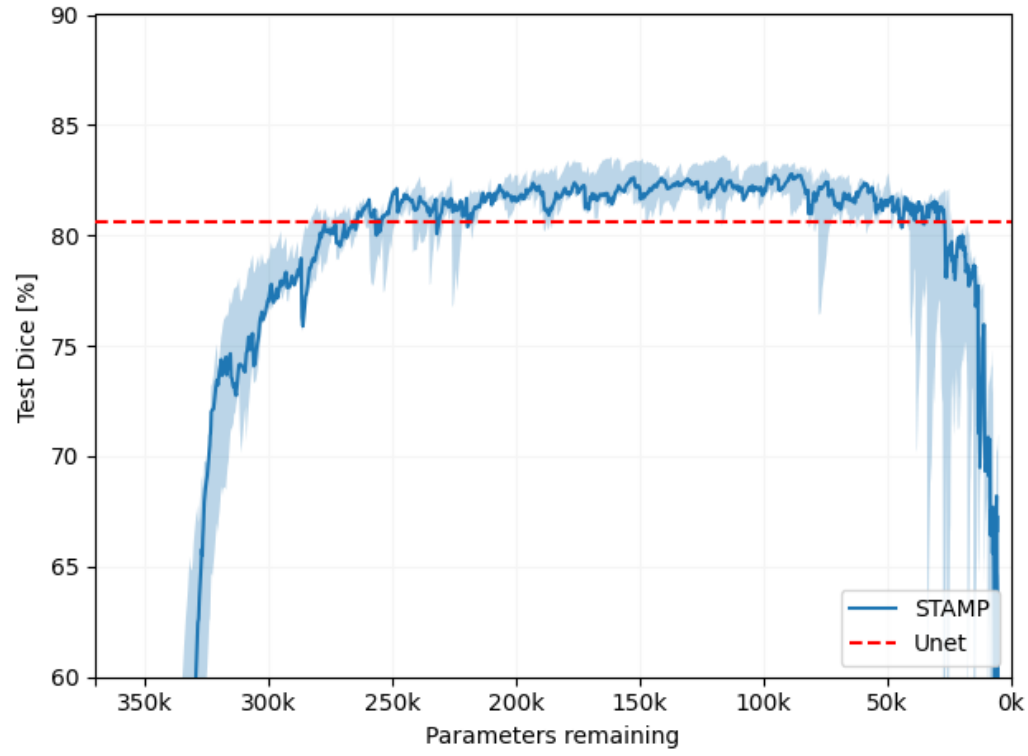

(a) Pruning vs. dense Unet

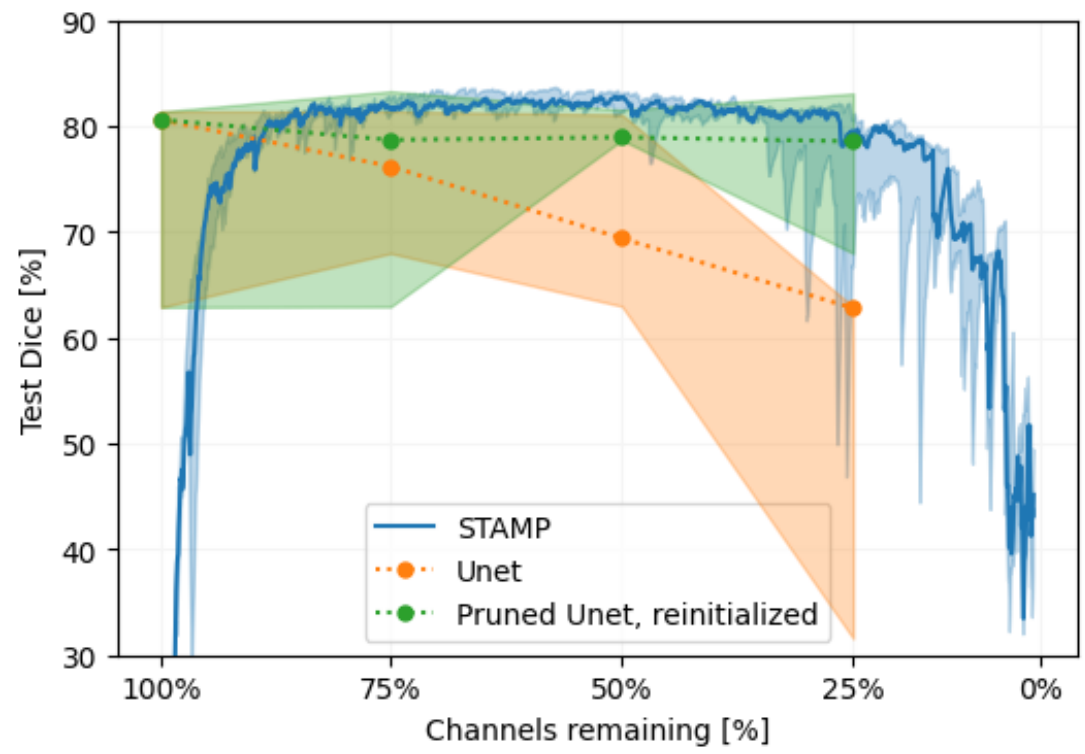

(b) Dense vs. scaled-down vs. reinitialized-pruned

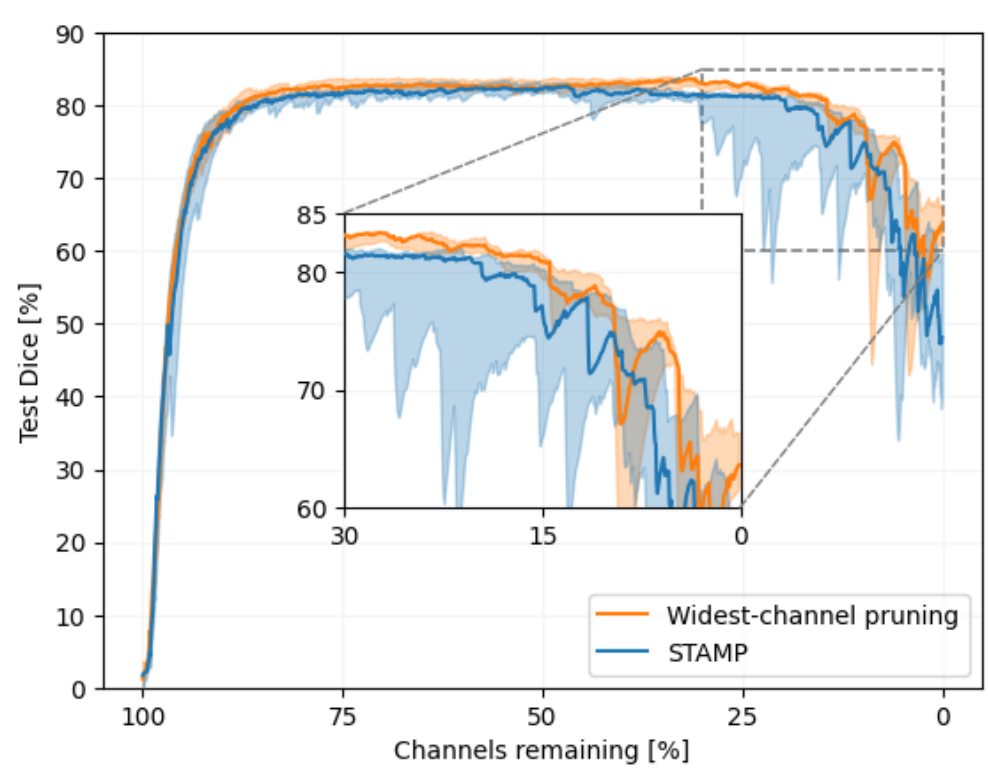

(c) Dice evolution of widest-block pruning and STAMP.

Fig. S2. Result figures on HarP with 50→220 train-test split. (a) Pruning compared to the baseline Unet. (b) Pruning compared to the baseline dense and linearly-scaled-down $\text{Unet}_{\{100\%,75\%,50\%,25\%\}}$ (orange) as well as equivalent-sized pruned networks after retraining from random initializations (green). (c) Dice evolution of STAMP based on channel activations compared to widest-block pruning. Curves show the median values, with min/max ranges of three repeated runs shaded.
